# Confirmation of the presence of *Anopheles stephensi* among internally displaced people’s camps and host communities in Aden city, Yemen

**DOI:** 10.1186/s12936-022-04427-9

**Published:** 2023-01-02

**Authors:** Richard Allan, David Weetman, Hendrik Sauskojus, Sophie Budge, Tarek Bin Hawail, Yasser Baheshm

**Affiliations:** 1 The MENTOR Initiative, Burns House, Harlands Road, Haywards Heath, RH16 1PG UK; 2 Liverpool School of Tropical Medicine, Pembroke Place, Liverpool, L3 5QA UK; 3 Ministry of Health in Yemen, National Malaria Control Programme, Aden, Yemen

**Keywords:** *Anopheles stephensi*, Conflict, Internally displaced people, Malaria, Temporary shelter, Urban vector, Water containers, Yemen

## Abstract

**Background:**

Declines in global malaria cases and deaths since the millennium are currently challenged by multiple factors including funding limitations, limits of, and resistance to vector control tools, and also recent spread of the invasive vector species, *Anopheles stephensi*—especially into novel urban settings where malaria rates are typically low. Coupled with general increases in urbanization and escalations in the number of conflicts creating rapid and unplanned population displacement into temporary shelter camps within host urban areas, particularly in the Middle East and sub-Saharan Africa, increased urban malaria is a major threat to control and elimination.

**Methods:**

Entomological monitoring surveys (targeting *Aedes aegypti*) of water containers across urban areas hosting internally displaced people (IDP) communities in Aden city, Yemen, were performed by The MENTOR Initiative, a non-governmental organisation. As part of these surveys in 2021 23 larvae collected and raised to adults were morphologically identified as *An. stephensi*. Twelve of the samples were sent to Liverpool School of Tropical Medicine for independent morphological assessment and genetic analysis by sequencing the ribosomal ITS2 region and the mitochondrial COI gene.

**Results:**

All twelve samples were confirmed morphologically and by sequence comparison of the single ITS2 and COI haplotype detected to the NCBI BLAST database as *An. stephensi*. Phylogenetic analysis with comparable COI sequences indicated close relationship to haplotypes found in Djibouti and Ethiopia.

**Conclusion:**

The study results confirm the presence of *An. stephensi* in Yemen. Confirmation of the species in multiple urban communities hosting thousands of IDPs living in temporary shelters with widescale dependency on open water containers is of particular concern due to the vulnerability of the population and abundance of favourable breeding sites for the vector. Proactive monitoring and targeted integrated vector management are required to limit impacts in this area of typically low malaria transmission, and to prevent further the spread of *An. stephensi* within the region.

## Background

*Anopheles stephensi* is an invasive disease vector, originally endemic in Asia and adept at transmitting malarial parasites *Plasmodium falciparum* and *Plasmodium vivax* [1, 2]. Native to south Asia and with long history of occurrence in the Persian Gulf states [3], the first record in Africa of *An. stephensi* was from Djibouti in 2012 [4], where it has since been associated with significant urban outbreaks of malaria in 2013 and 2014, and continuously increasing annual urban malaria case numbers through to 2017 [5, 6]. Subsequent discoveries in four countries, three of which host  > 10.5 million conflict and disaster displaced people in camps and urban settings [7, 8]; Ethiopia [9], Sudan [10] in 2019 in Somalia [11] and in 2020 in Somaliland [12] (since confirmed in multiple sites in each country [13–15]), suggest *An. stephensi* is becoming established in the region and could spread further, with potentially serious consequences for malaria control and elimination [3, 15].

Unsurprisingly, the introduction of *An. stephensi* into the Horn of Africa has generated substantial global concern and calls for action [16]; this is primarily because of its potential to elevate malaria in densely populated urban environments where transmission has typically been considered lower than in rural communities [17, 18]. Unlike most *Anopheles* species which tend to lay eggs in natural water sources and are relatively rarely found in urban settings, *An. stephensi*, in the same manner as *Aedes aegypti,* frequently lay their eggs in man-made water containers, including waste or polluted waters [19, 20]. *Anopheles stephensi* are thus well adapted to breeding sites created by urbanization and thrive in such settings [1, 2]. Whilst the spread of *An. stephensi* in the Horn of Africa has attracted much recent attention, its invasion presents a threat to any countries with large populations located within habitats permissive for successful establishment [3]. In sub-Saharan Africa and countries within the Middle East an increasing proportion of populations live in densely populated cities with insufficient water, sanitation, and hygiene (WaSH) and poor environmental hygiene, conditions in which *An. stephensi* may thrive and elevate malaria transmission.

Since 2000, the global Roll Back Malaria partnership has made significant progress in malaria reduction through a combination of scaling up access to insecticidal mosquito nets or indoor residual spraying and in access to effective rapid diagnostic testing and drug therapies. Global malaria deaths declined steadily from 896,000 to 558,000 by 2019 [21]. However, over the last five to six years malaria deaths have increased again significantly to an estimated annual 627,000 by end-2020 [21]. This rise has occurred in parallel with a dramatic escalation in the number of conflicts and the number of internally displaced people (IDPs). By end-2021, an unprecedented 53.2 million people across 59 countries and territories were displaced as a result of conflict and violence—an increase of 5.8 million from 2020 [22]. The combination of increasing conflict, the destruction of urban areas and population displacement into urban settings appear inextricably linked to rises in cases of vector-borne diseases [23, 24]. Violence and widespread destruction of public and private infrastructure can mean populations have limited access to basic services such as healthcare, WaSH and education [23]. This logically increases the risk of vector-borne diseases. Further, research has shown that mass population displacement can significantly increase the incidence of severe malaria [24, 25] and cause excess mortality and morbidity among displaced communities [26, 27], particularly when they are immunologically vulnerable [25]; displaced children also appear at significantly higher risk than their non-displaced counterparts [28–30]. This bears concern for outbreaks in densely populated, fragile urban areas [31].

The ongoing civil war in Yemen began in 2014. At the end of 2021 almost 21 million people were in need of humanitarian assistance, and 4.3 million people had been internally displaced, making Yemen one of the most acute conflict based crises in the world. Many IDPs are hosted in urban settings from north west to the south west [32]. In addition to ongoing conflict and mass population displacement, many people are at risk of vector-borne diseases. Malaria, dengue fever, leishmaniasis and other vector borne diseases, are endemic across certain areas of Yemen. Whilst epidemiological reporting since 2014 has been limited, concerning changes in the burden and spread of dengue fever in Alhodeidah, Taiz, Aden and Al-Mukalla [33] have been reported, together with outbreak reports of dengue fever in Taiz city [34] and the capital, Aden, where annual cases have been rising.

Much of the country, inland from the coastal zones, is malaria free. Areas of relatively high transmission are largly concentrated in the lowland western coastal zones and a few southern coastal areas. The inland areas neighbouring the coastal zones, are generally higher altitude and many of the countries urban centres are sited in these higher altitude locations. In these, malaria transmission ranges from medium to very low transmission. However, due to the country population’s geographic distribution, and concentration towards the coastal zones, up to 78% live in malaria risk areas. Roughly 25% are located in high risk areas (> 1 cases in 1000), mainly concentrated down the western side (Tehama Region) of the country. The major vectors are reported to be *Anopheles arabiensis, Anopheles culicifacies,* and *Anopheles sergentii.* The annual malaria case load reported by functioning health facilities is around 143,000 (as of 2017) with fewer than 40 reported deaths, but many cases are believed to occur in the coastal communities most directly affected by conflict, with least access to functioning health facilities, and are unreported. Over 99% of all reported cases are caused by *P. falciparum* [35]. Historically, Aden city was considered to have very low risk of malaria transmission, but since the begining of the current war, WaSH conditions have deteriorated across many urban areas and annual malaria and dengue case reports from health facilities across Aden governorate have been increasing to concerning levels. MoH has integrated entomological surveillance activities for *Aedes* and *Anopheles* mosquitoes, and vector control campaigns and health facility support have been scaled up in some urban areas.

The MENTOR Initiative (MI), an international non-governmental organisation, in partnership with the National Malaria Control Programme (NMCP) and the World Health Organization (WHO) supports efforts to control vector-borne diseases in Aden. As part of its operational research work*,* MI conducts entomological monitoring surveys of water containers across IDP camps [36] and their surrounding host urban areas of Aden city, to detect and collect mosquito pupae. Pupae are then cultured in an insectory to morphologically identify emerging adult mosquitoes. In 2021, as part of this entomological monitoring, adults emerging from pupae were identified morphologically as *An. stephensi.* This study reports PCR validation of these collections, confirming the presence of the vector in Yemen. This report also contributes to those of others registered in the WHO Threats Map, enabling mapping the vector’s distribution across the country [11]. Findings bear significance not just for the identification of invasive vectors and the expanding malaria risk across urban settings, but also for the fact that this vector was identified in an urban community hosting thousands of persons displaced within the country who are vulnerable to infection.

## Methods

### Entomological surveillance

The programme in Yemen ran between the months of February to December 2021, and entomological surveys were carried out each month from June and October of the same year, covering 36 accessible IDP sites and their urban host communities in Al-Buraiqha (6), Dar Saad (15), Al-Sheikh Othman (5), and Al-Mansoorah (10) districts of Aden city (Table 1). Entomological survey locations were chosen at random from a list of all accessible IDP camps and host communities in Aden, within which a total of 500 households were randomly selected and surveyed.

**Table 1:** Neighbourhoods in Aden City selected for entomological household surveys

Aden city districts				
	Dar Saad	Al-Mansoorah	Alshiekh Othman	Al-Buraiqha
Neighbourhoods Surveyed	Al-Lahoom	Beer Fadahl	Al-Memdahra Alqadeem	Al-Qayedah Al-Russia
	Al-Basateen Block 1	Al-Kahera Al-Magmaa	Kood Al-Othmani	Al-Hofraha
	Al-Basateen Block 7	Al-Kahera Al-Shoolah	Al-Sysban	Al-Faresi-Dabash Camp
	Al-Basateen Block 9	Hashed	Al-Mahareeq	Al-Faresi
	Al-Basateen Block 21	Al-Kahera Al-Masbah	Al-Saylaha	Al-Kaysaaha
	Al-Basateen Block 22	Wadae Hadad		Al-Shab IDP Camp
	Al-Basateen Block 23	Al-Durain		
	Al-Basateen Block 24	Al-Sharooq Squire		
	Al-Gharbiah	Al-Taqnia City		
	Al- Sharqiah	Block 30		
	Hosh Derham IDP Camp	Block 36		
	Al-Sudei IDP Camp			
	Al-Sudei IDP Camp			
	Suidi Institute IDP Camp			
	Zahra Khalil IDP Camp			
	Amar Bin Yasser IDP Camp			

After explaining the procedure and intent behind the survey, the head of each household was asked for their consent in participating. If they agreed, all water containers and any other receptacles holding water and suitable for mosquito breeding were assessed for mosquito larvae and pupae, which were collected using ladles and brought back to MI’s insectary in Aden. After rearing the larvae and pupae into adult mosquitoes, species were determined using a Bresser Researcher ICD LED 20X-80X Stereo Microscope and 12 of 23 mosquitoes of interest were sent to the Liverpool School of Tropical Medicine for further evaluation. The remaining 11 samples were stored in Aden for Ministry of Health for future examination.

### Laboratory confirmation

The sample of twelve adult female mosquito specimens were identified morphologically, with particular focus on the most notable diagnostic characters—the two broad apical bands, and speckled third segment on the maxillary palps and banding patterns on wing vein one [37]. DNA was extracted from two legs per specimen using a simple sodium chloride-tris–EDTA (STE) boiling method. Two mosquito legs were incubated at 95 ℃ for 90 min in 20 µl of STE buffer, 0.1 M NaCl, 10 µmol TrisHCl pH 8.0 and 1 µmol EDTA pH8.0, followed by internal transcribed spacer (ITS2) sequencing using generic *Anopheles* primers [38]; and cytochrome oxidase I (COI) sequencing using primers LC1490 and HC2183 [39]. The ITS2 polymerase chain reaction (PCR) contained 2.5 µl of 10 × Fermentas Dream Taq buffer with 20 nM MgCl, 0.5 µl of 10 µM dNTPs, 1.5 µl of each 10 µmol ITS2A and ITS2B primers, and 0.1 µl of Fermentas DreamTaq at 5units/µl (Thermo Fisher cat no. EP0711), 2 µl of extracted DNA—made up to a final volume of 25 µl with molecular-grade water. The thermocycling conditions were: 95 ℃ for 5 min, 35 cycles of denaturation at 95 ℃ for 30 s, annealing at 50 ℃ for 30 s, extension at 72 ℃ for one minute, and a final extension of 72 °C for 10 min. The COI PCR reaction contained 4.0 µl of 10 × Fermentas DreamTaq buffer with 20 nM MgCl, 0.8 µl of 10 µM dNTPs, 1.6 µl of each 10 µM LC1490 and HC2183 primers, and 0.16 µl of Fermentas DreamTaq at 5units/µl (Thermo Fisher cat no. EP0711), 2 µl of extracted DNA—made up to a final volume of 40 µl with molecular-grade water. The thermocycling conditions were: 95 ℃ for two minutes, 35 cycles of denaturation at 95 ℃ for 30 s, annealing at 52 ℃ for 30 s, an extension at 72 ℃ for 50 s, and a final extension at 72 ℃ for two minutes. The amplified fragments from both PCRs were visualised by electrophoresis on 2% agarose gel. The PCR products were purified using a Qiagen PCR purification kit (Qiagen, cat no. 28104). Samples were sent for sequencing, using both the forward and reverse primers.

## Results

**Fig. 1:**
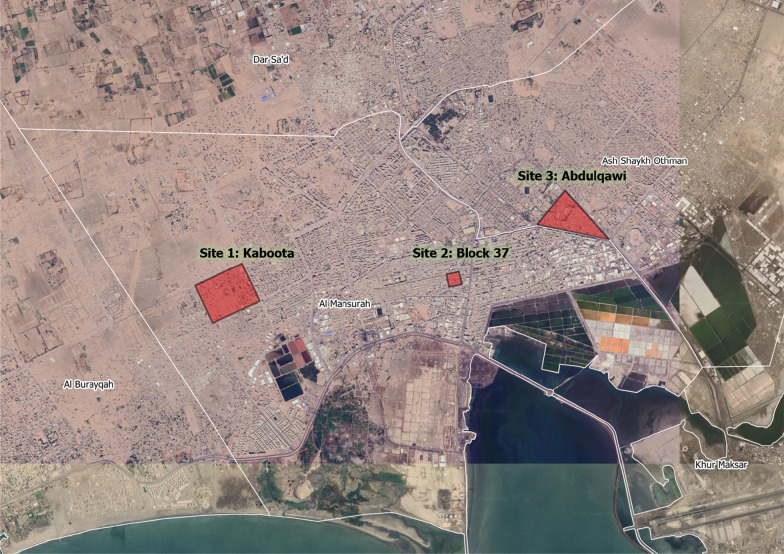
Map of locations in Aden city in which 23 identified *An. stephensi* specimens were found: September–October 2021

In total, 1003 mosquito pupae were collected during the five rounds of entomological surveillance in Al-Buraiqha, Dar Saad, Al-Sheikh Othman, and Al-Mansoorah, resulting in 847 emergent adult moquitoes. Of these, 824 were morphologically identified as *Aedes* mosquitoes and 23 as *An. stephensi* (Table 2). The geographical locations of the pupae of the *An. stephensi* mosquitoes are described in Table 3, and shown in Fig. 1. These pupae were found in car tyres, a bucket, a plastic water tank and a jerry can (Table 3).

**Table 2:** Number of pupae collected during entomological surveys in IDP hosting sites in Al-Buraiqha, Dar Saad, Al-Sheikh Othman, and Al-Mansoorah and number of adult *Aedes*/*An. stephensi* emerged

Month	Round	No. pupae collected	No. *Aedes* emerged	No. *An. stephensi* emerged
June	R1	91	75	0
July	R2	303	230	0
August	R3	217	181	0
September	R4	184	158	2
October	R5	208	180	21
Total	5	1003	824	23

**Table 3:** Geographical location and habitats of the 23 *An. stephensi* specimens in Yemen: September–October 2021

District	Neighbourhood/IDP site	Coordinates	Container type	#Female	#Male	Month
Al-Mansoorah	Kaboota	12°51ʹ35.88ʺN 44°57ʹ18.71"E	Plastic tank	1	–	September
Al-Mansoorah	Block 37	12°51ʹ34.31ʺN 44°59ʹ13.05ʺE	Jerrycan	1	–	September
Al-Sheikh Othman	Abdulqawi	12°51ʹ59.78ʺN 45°00ʹ23.96ʺE	Car tyre	4	7	October
Al-Sheikh Othman	Abdulqawi		Car tyre	3	6	October
Al-Sheikh Othman	Abdulqawi		Bucket	1	–	October

**Fig. 2:**
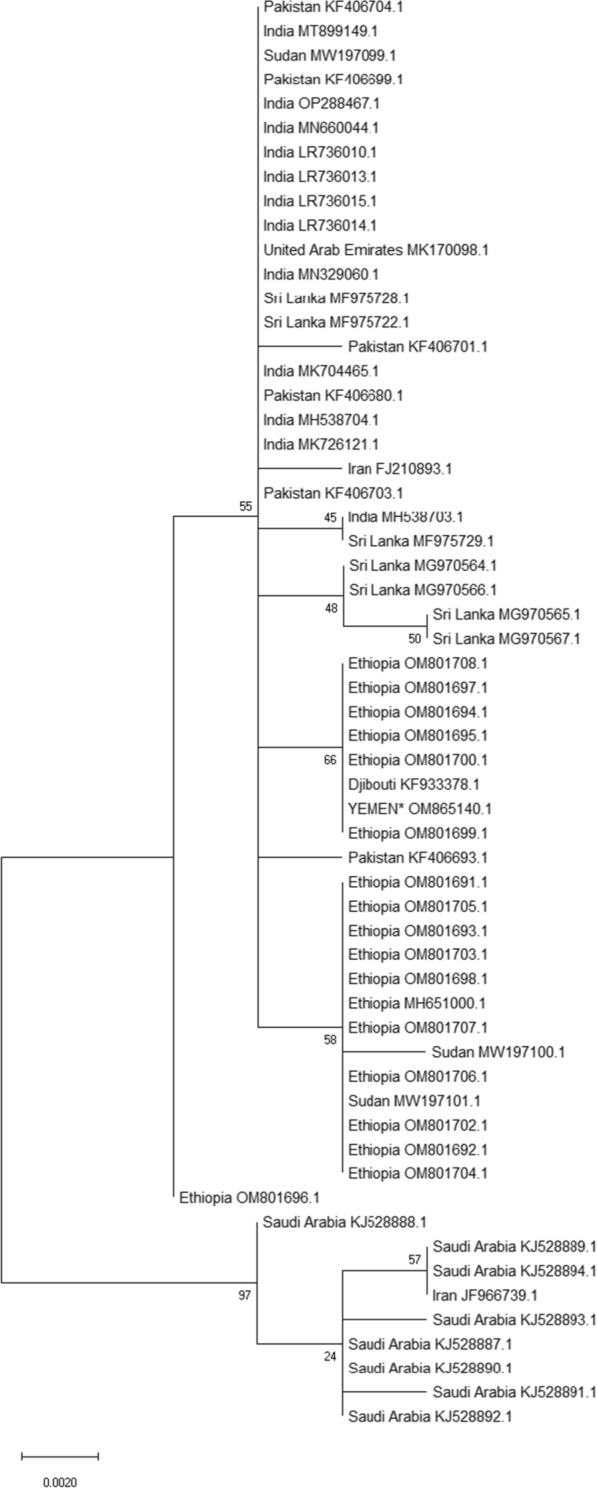
Maximum-likelihood phylogeny comparing the COI sequence from Yemen (highlighted) with all comparable sequences downloaded from GenBank. Scale bar indicates nucleotide substitutions per site

All 12 ITS sequences (472 nucleotides in alignment) were identical and using NCBI BLAST search, all high confidence matches were to *An. stephensi* (most 99.5–100% depending on the length of the target sequence; GenBank accession number: OM869998). Sequences were identical to the major branch of a previously published ITS phylogeny, which included samples from across the known range of *An. Stephensi* [1]. Eleven of the 12 samples produced high quality COI sequences and all were of an identical 536 nucleotide haplotype (GenBank accession number: OM865140). BLAST searching of the haplotype sequence identified 59 *An. stephensi* COI sequences with at least 90% shared coverage after removal of colony samples. Sequences were aligned, trimmed to 449 nucleotides to give a uniform length (corresponding to *An. stephensi* mitochondrial genome positions 1568-2016) and a maximum likelihood tree (using the Tamurai-3-parameter model) constructed in MEGA X [40] with 1000 bootstraps (Fig. 2).

**Fig. 3:**
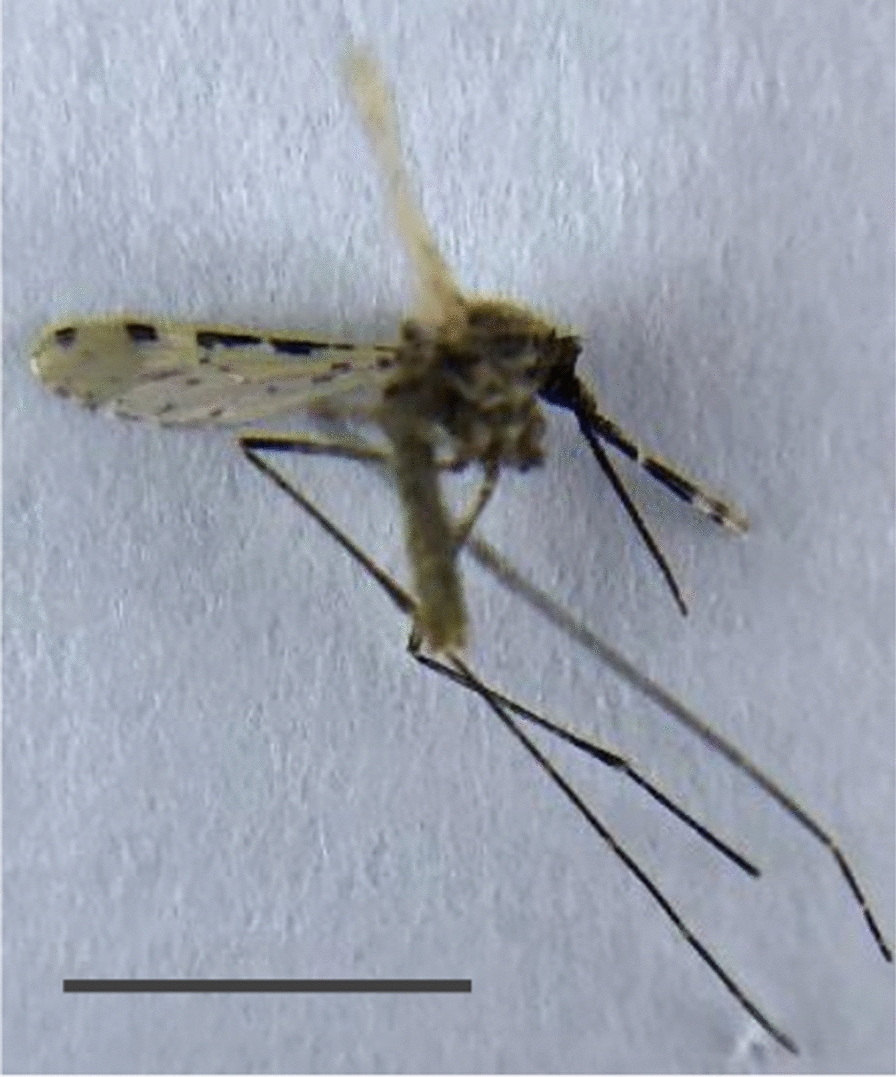
Female *An. stephensi* specimen sample identified in Aden, September, 2021. The scale bar represents approximately 5 mm

The haplotype from Yemen grouped with the sole sequence from Djibouti and several from Ethiopia [41], with reasonable bootstrap support, suggesting possible commonality of origin. However, it should be noted that this clustering was based on a single shared nucleotide variant, which separated the haplotypes on this node from others on the major branch. Moreover, although data are increasing, the diversity of COI-sequenced samples available remains quite limited, precluding confident identification of origins at present. The photograph in Fig. 3 shows one of the identified adult *An. stephensi* specimens, including the notable diagnostic characteristics of banding patterns on the maxillary palps and wing vein one.

## Discussion

The results presented provide morphological identification and genetic characterization of *An. stephensi* in southern Yemen within an urban setting hosting IDPs. The vector exists in Kuwait [42], Saudi Arabia [43], The United Arab Emirates and Qatar [11]; and the findings presented here confirms that *An. stephensi* is now also established in southern Yemen, alongside recent identification in the Horn of Africa [4, 9, 16]. Later reports, based on morphological identification of *An. stephensi,* listed on the WHO Threat Map between October 2021 and March 2022, register the vector in Aden city, Al Hodayda (situated to the far east on the Red Sea) and Hadramout (north east) [11]. Indeed, *An. stephensi* appears to now be well-established within the country and likely exists in many more locations than is currently reported. While a nationwide survey of Yemen would be desirable to map the vector’s spread across the country, surveys can be logistically difficult due to limitations in funding and human resource capacity, as well as restrictions to access due to security conditions. However, local surveys, where possible, particularly in the western part of Sanaa governorate, including Tihama region, would help establish an accurate distribution map for *An. stephensi* in these parts of Yemen and would also aid in identifying the breeding sites most conducive to larvae development prior to the implementation of any vector control methods [44, 45].

An important point in the spread of this highly invasive vector to Yemen lies in the context of its identification site—that is, within a conflict-affected, fragile community hosting large numbers of IDPs with widescale destruction of both public and private infrastructure and services. The movement of immunologically naïve individuals from areas of low to high risk can contribute to the disease impact of an already highly invasive vector where they are more likely to develop severe disease [27–29]. In this study setting of a host community, and in related fragile settings such as camps for IDPs, an already vulnerable population face increased mortality risk [27–30] particularly from *P. falciparum* malaria which is often rapidly fatal during the acute phase of an emergency [30].

The threat of *An. stephensi* is not an isolated one given its similarities in urban ecology to *Aedes* mosquitoes which transmit four major disease pathogens (dengue virus, yellow fever virus, chikungunya virus, and Zika virus). Prior to vector control programmes for *Anopheles* species and particularly *An. stephensi*, implementors and local authorities must seek to understand how they might integrate programmes into current management strategies (including those designed to manage *Aedes* species) to deliver effective, pragmatic, and timely control. Current challenges surrounding integrated vector management programmes and those faced by implementing bodies and relevant organisations, as well as the lessons that can (and should) be learned from previous and current *Aedes* control programmes are described elsewhere [46].

## Conclusion

This study confirms the presence of *An. stephensi* in a conflict-affected, fragile urban setting in Yemen. The *An. stephensi* detected showed similar breeding sites to those documented in the Horn of Africa [4, 9, 10, 12], and highlights the importance of integrating WaSH measures with larval control, given a reliance on open water containers. Further detection and genomic analyses are required to reveal the pattern and direction of spread to inform integrated vector control programmes aiming to control *An. stephensi* in urban settings in Yemen and to prevent further spread across the region—particularly within poor, vulnerable urban communities.

## Data Availability

The datasets supporting the conclusions of this article are included within the article and its supplemental files. Sequences have been submitted to NCBI Genbank database.

## Abbreviations

COI: Cytochrome c oxidase subunit I
IDP: Internally displaced people
IRS: Indoor residual spraying
ITS: Internal transcribed spacer
MI: The MENTOR Initiative
mtDNA: Mitochondrial DNA
PCR: Polymerase chain reaction
UNHCR: United Nations High Commissioner for Refugees
WaSH: Water, sanitation, and hygiene
